# Reversible Reactions of Nitric Oxide with a Binuclear Iron(III) Nitrophorin Mimic

**DOI:** 10.1002/chem.202302860

**Published:** 2023-11-21

**Authors:** Vinay K. Sharma, Azad Saini, Natalia Fridman, Harry B. Gray, Zeev Gross

**Affiliations:** [a] Schulich Faculty of Chemistry, Technion – Israel Institute of Technology Institution, Haifa 32000 (Israel); [b] Beckman Institute, California Institute of Technology, Pasadena, California 91125 (USA)

**Keywords:** corrole, dimer, heme, nitric oxide, reversible reaction

## Abstract

Construction of functional synthetic systems that can reversibly bind and transport the most biologically important gaseous molecules, oxygen and nitric oxide (NO), remains a contemporary challenge. Myoglobin and nitrophorin perform these respective tasks employing a protein-embedded heme center where one axial iron site is occupied by a histidine residue and the other is available for small molecule ligation, structural features that are extremely difficult to mimic in protein-free environments. Indeed, the hitherto reported designs rely on sophisticated multistep syntheses for limiting access to one of the two axial coordination sites in small molecules. We have shown previously that binuclear Ga(III) and Al(III) corroles have available axial sites, and now report a redox-active binuclear Fe(III) corrole, (**1-Fe**)_2_, in which each (corrolato)Fe(III) center is 5-coordinate, with one axial site occupied by an imidazole from the other corrole. The binuclear structure is further stabilized by attractive forces between the corrole π systems. Reaction of NO with (**1-Fe**)_2_ affords mononuclear iron nitrosyls, and of functional relevance, the reaction is reversible: nitric oxide is released upon purging the nitrosyls with inert gases, thereby restoring (**1-Fe**)_2_ in solutions or films.

Heme proteins perform a myriad of vital functions, notably including the transport and/or activation of O_2_ and NO.^[1–4]^ The prosthetic groups of O_2_-binding myoglobin and NO-transferring nitrophorin are similar in that each contains a 5-coordinate heme with an axial histidine ligand, as well as a vacant *trans* axial site for binding small molecules.^[2,5–9]^ Design of synthetic systems in which one and only one histidine (or imidazole) is bound in a protein-free environment is challenging, owing to the tendency of both iron(II) and iron(III) porphyrins to be coordinatively saturated.^[10]^ Architecture-like synthesis is one way to obtain coordinatively unsaturated complexes, as exemplified by tethering an imidazole group to a porphyrin core or by sterically blocking a coordination site (a and b in Scheme 1, respectively).^[10–12]^ Mimicking the Fe/Cu active site of cytochrome c oxidase requires an even more elaborate architecture, namely 4 tethered imidazole units of which only one coordinates to the heme along with three others for Cu binding (c in Scheme 1).^[13–16]^

The motivation of the present investigation was our finding that the binuclear complexes (**1**-Ga)_2_ and (**1**-Al)_2_ (d in Scheme 1) remain 5-coordinate even in the presence of strongly donating ligands.^[17]^ We reasoned that O_2_ and/or NO might bind to the vacant axial site in the redox-active Fe(III) analogue (**1**-Fe)_2_; and, indeed, the NO reaction with (**1**-Fe)_2_ is reversible, in contrast to the irreversible reaction of NO with monomer **2**-Fe(N-imid)_2_.

The bis-pyridine complex **2**-Fe(py)_2_ has been previously reported,^[18]^ but that is not the case for **2**-Fe(N-imid)_2_ which has now been prepared (Figure 1a). Both the axial and equatorial Fe–N bond lengths are shorter in the latter complex: Fe-N_imid_ = 1.988(4) Å vs. Fe-N_py_ = 2.030(5) Å; and (average) Fe-N_corrole_ = 1.868(5) and 1.894(5) Å for **2**-Fe(imid)_2_ and **2**-Fe(py)_2_, respectively. Of interest is that treatment of either **2**-Fe(N-imid)_2_ or **2**-Fe(py)_2_ with NO led to the formation of an iron nitrosyl, designated **2**-Fe(NO). Apparently, both complexes spontaneously lost one axial ligand in solution and another one upon NO binding (Figure S8 and S10), chemistry consistent with earlier studies.^[18–20]^ Once formed, **2**-Fe(NO) did not release NO when exposed to nitrogen or air.

Iron insertion into the C_10_-imidazole-substituted corrole proceeded in 85% chemical yield. The product, an iron(III) dimer (**1**-Fe)_2_ (Scheme 1d), was fully characterized by UV-vis spectroscopy, mass spectrometry, NMR, cyclic voltammetry, and X-ray crystallography. Crystal structure analysis confirmed that (**1**-Fe)_2_ (Figure 1b) is very similar in structure to the Ga(III) and Al(III) analogues.^[17]^ The coordination geometry of each iron center in (**1**-Fe)_2_ is square pyramidal, with an out-of-corrole N_4_ plane displacement of 0.38 Å. The deviation, which is 0.08 Å greater than that for deoxy Mb (0.30 Å)^[21]^ is comparable to those of the Ga(III) and Al(III) analogues. The average Fe-N_corrole_ bond [Fe–N_c_ = 1.893(8) Å in (**1**-Fe)_2_] is 0.14 Å shorter than that in deoxy Mb,^[21]^ as well as in Ga(III) and Al(III) analogues,^[17]^ but slightly longer than in **2**-Fe(N-imid)_2_. Comparison of average Fe–N axial bond lengths between (**1**-Fe)_2_ and **2**-Fe(imid)_2_ revealed that the former is longer, 2.099(5) Å vs 1.988(4) Å. The mean plane separation (MPS) of the two corrole subunits in (**1**-Fe)_2_ is 2.935 Å, somewhat shorter than in the analogous (**1**-Ga)_2_ and (**1**-Al)_2_ and more so relative to co-facial porphyrins.^[17]^ This suggests that the attractive π-π interaction between the aromatic rings in (**1**-Fe)_2_ is exceptionally strong, a hypothesis that is further supported by electrochemistry (see below). Additional X-ray crystallographic data for the iron complexes are in Table S1 & S2. High-resolution mass spectrometry confirmed that (**1**-Fe)_2_ remains dimeric even in the gaseous state, with m/z=1498.0405 for C_68_H_22_F_20_Fe_2_N_12_ (Figure S3). Regarding the electronic structure, preliminary investigations disclose that it is EPR-silent at temperatures down to 5 K and according to SQUID has a magnetic moment of 2.9 μ_B_ per iron atom at 300 K (Figure S11).

The structural integrity of (**1**-Fe)_2_ was examined in solution by UV-vis, electrochemistry, and NMR. The data confirm that (**1**-Fe)_2_ remains binuclear even in very dilute solution (7 μM - 5 mM). However, reactions occur when (**1**-Fe)_2_ and **2**-Fe(N-imid)_2_ are treated with increasing amounts of N-methylimidazole (N-imid). The initial spectra of the two complexes were quite similar in solution, (bis-amine)Fe(III) corroles are in active equilibrium between the 5- and 6-coordinate states.^[22]^ Transformation to bis-N-imid iron(III) coordination (documented by 590–650 nm absorptions) produced 30% and 100% increases in the Soret intensity for the mono- and binuclear complexes, respectively (Figure S2). This finding is consistent with a more drastic change for (**1**-Fe)_2_ from binuclear (pure 5-coordinate) to mononuclear (6-coordinate).

Cyclic voltammetry (CV) was employed to investigate inter-subunit interactions upon oxidation and reduction of (**1**-Fe)_2_. Anaerobic CV data collected for (**1**-Fe)_2_ (0.5 mM, in PhCN containing 0.1 M tetrabutylammonium perchlorate) revealed two reduction and two oxidation processes. The oxidation waves were reversible, with half-wave potentials (E_1/2_) of 0.62 and 0.83 V (Figure 2a). This finding of two reversible corrole-based oxidation reactions indicates that (**1**-Fe)_2_ remains intact. Also notable is the 0.21 V redox potential difference, larger than for the analogous Ga(III) complex (**1**-Ga)_2_ (ΔE_1/2_ = 0.17 V),^[17]^ consistent with the earlier discussed smaller interplanar distance in (**1**-Fe)_2_.

In contrast, only the second reduction of (**1**-Fe)_2_ is reversible. Examination of the same redox event with the mononuclear **2**-Fe(py)_2_ [**2**-Fe(N-imid)_2_ is sparingly soluble in PhCN] reveals that its E_1/2_ value is 140 mV less negative than for the binuclear complex. This is attributable to the *meso*-imidazole substituent in (**1**-Fe)_2_, which is less electron withdrawing than the −C_6_F_5_ group of **2**-Fe(py)_2_. However, the difference is much larger for the first reduction reaction: the anodic peaks are −0.9 and 0.46 V for (**1**-Fe)_2_ and **2**-Fe(py)_2_, respectively (Figure 2b and S4). This 440-mV difference is clearly attributable to the axial imidazole being a stronger donor than pyridine. Taken together, these results suggest that reduction of the binuclear complex triggers dissociation to the mononuclear form (Figure 2c). Additional support for this proposal is that the reoxidation peaks of the products obtained by single electron reduction are virtually identical for (**1**-Fe)_2_ [E_pc_ = −0.38 V, Figure 2b] and **2**-Fe(py)_2_ [E_pc_ = −0.37 V, Figure S4]. The outcome is that the characteristics of (**1**-Fe)_2_, featuring iron centers with imidazole and an empty binding site trans to it, are lost upon its reduction to the iron(II) oxidation state. This largely reduced affinity to external ligands is consistent with the two studies that reported the isolation of iron(II) corroles.^[42,43]^

As nitrophorins have been shown to bind NO in both the Fe(II) and Fe(III) states,^[23–26]^ our focus turned to the study of its reactions with (**1**-Fe)_2_. We note that high affinity NO binding has traditionally focused mainly on ferrous model complexes,^[11,26–28]^ not at least because of difficulties of obtaining stable ferric nitrosyl complexes.^[29]^ Treatment of (**1**-Fe)_2_ with NO gas in anaerobic tetrahydrofuran (THF) solution immediately converted the initial brick red solution to deep orange, accompanied by a blue shift in Soret absorption (403 to 380 nm) and appearance of a new feature at 504 nm, as well as disappearance of the 724 nm band (Figure 3a). Clear indication for formation of a nitrosyl complex was obtained by recording the CV of (**1**-Fe)_2_ in THF solution with dissolved NO gas. A 1.1 V shift in the first reduction peak was observed (Figure 3b), similar to that recorded upon **2**-Fe conversion to **2**-Fe(NO) (Figure S7), in agreement with a prior report.^[31]^ Importantly, N_2_ purging the orange solution restored the original brick-red color and electronic spectrum (Figure 3a, blue trace), thereby indicating *reversible* NO reactivity. In contrast, **2**-Fe(NO) did not release NO upon purging and even not on chromatographic columns.

More evidence was obtained by recording the 19F NMR spectrum of (**1**-Fe)_2_ in THF-*d*_*8*_ (Figure 4b). Consistent with a dipolar (=pseudo-contact=through space) component being dominant, as in paramagnetic NMR spectra of intermediate spin iron(III) porphyrins,^[32–35]^ all ^19^F resonances of (**1**-Fe)_2_ were shifted in the same direction. That was in the order of *ortho*->*meta*-> and *para*-F: by 41, 6, and 3 ppm, respectively, relative to the diamagnetic analogue (**1**-Ga)_2_ (Figure 4a). The full width at half maximum (FWHM) of the respective resonances also are in the same order: 1.19, 0.14, and 0.11 ppm. Consistent with a binuclear structure (Figure 1b) is the clear separation between the *ortho*- and *meta*-F resonances in each of the four identical C_6_F_5_ groups.

Upon NO(g) purging of the same solution, the *ortho*-F resonances shifted from −99 to −140 ppm, followed by *meta*-F from −158.7 to −163.2 ppm, and *para*-F from −154.0 to −155.8 ppm (Figures 4b and 4c). These changes clearly point toward a change from para- to diamagnetic complex, consistent with the formation of either a mononuclear **1**-Fe(NO) (similar to **2**-Fe(NO) whose spectrum is depicted in Figure 4d) or a binuclear **1**-Fe(NO)_2_ complex. Evidence in favor of the first possibility comes from comparison of the spectra in Figures 4a and 4c: the differences between the two kinds of *ortho*-F and *meta*-F atoms in (**1**-Ga)_2_ are about 2 times larger than in the product obtained from combination of (**1**-Fe)_2_ with NO. This finding suggests that NO coordination to (**1**-Fe)_2_ triggers dissociation to mononuclear units via detachment of the imidazole that holds them together, consistent with the very strong trans effect of nitric oxide.^[36,37]^ Examination of the same solution by ^1^H NMR provided even stronger evidence for the formation of **1**-Fe(NO). Unlike (**1**-Ga)_2_ but much like its free base **1**-H_3_,^17^ all the *β*-pyrrole C–H and imidazole N–H resonances appeared in the ‘normal’ aromatic region. This is consistent with the formation of a mononuclear complex in which these H atoms do not experience any diamagnetic current effect from another corrole. A unique finding was that **1**-Fe(NO) is only stable in the presence of excessive amounts of dissolved NO(g). Although purging with N_2_(g) led to a very complex NMR spectrum, pure (**1**-Fe)_2_ was isolated following column chromatography. In contrast, NO binding to **2**-Fe(N-imid)_**2**_ was irreversible; notably, product **2**-Fe(NO), which was isolated and fully characterized (Figures 4d and S6–9)^[18,39]^ remained unchanged upon purging with either inert gases or air or upon column chromatography treatment. A plausible explanation for this difference is that attractive forces between macrocyclic subunits (<3 Å distance and largely separated oxidation potentials, see above) enhance the binding of imidazole associated with formation of the binuclear complex.

The robustness of the reversible NO reaction with (**1**-Fe)_2_ was tested by recording 8 cycles of NO(g) treatment followed by Ar(g) purging (virtually identical results were obtained with N_2_(g); and both under ambient light and in the dark). Changes in both the color and λ_max_ of the main absorption were followed (Figure 5a), confirming robust reversible N O reactivity. As NO binding to solid materials is important in many applications,^[40]^ a solution of (**1**-Fe)_2_ in CH_2_Cl_2_ was drop-cast on a platinum surface of the IR device (Figure 5b, black trace). Repeating the same procedure using a freshly prepared NO-purged solution revealed a new N–O stretching band at 1795 cm^−1^ (Figure 5b, red trace). This band is attributed to the presence of an iron nitrosyl, and its frequency closely resembled the N–O stretching frequency of **2**-Fe(NO) (1800 cm^−1^, Figure S9, red trace), as well as other monomeric Fe(III) corrole and porphyrin complexes.^[19,29,39,41]^ Notably, the N–O stretching band disappeared when the modified surface was purged with Ar(g) in the case of (**1**-Fe)_2_ (Figure 5b, blue trace), while this phenomenon did not occur for **2**-Fe(NO).

In summary, we have demonstrated reversible NO reactivity with (**1**-Fe)_2_ in which a *meso*-imidazole moiety of one monomeric unit is coordinated to the iron in the other unit. Nitric oxide binding to one of the vacant axial coordination sites in the binuclear complex occurs even in the ferric state, which triggers dissociation to mononuclear iron-nitrosyls. As NO concentrations are lowered, repopulation of (**1**-Fe)_2_ is favored, owing to the strong dimerization driving forces. The discovery of reversible nitric oxide reactivity with (**1**-Fe)_2_ paves the way for development of advanced sensing systems and real-time NO concentration monitoring devices.

## Supplementary Material

supporting information

## Figures and Tables

**Figure 1.**
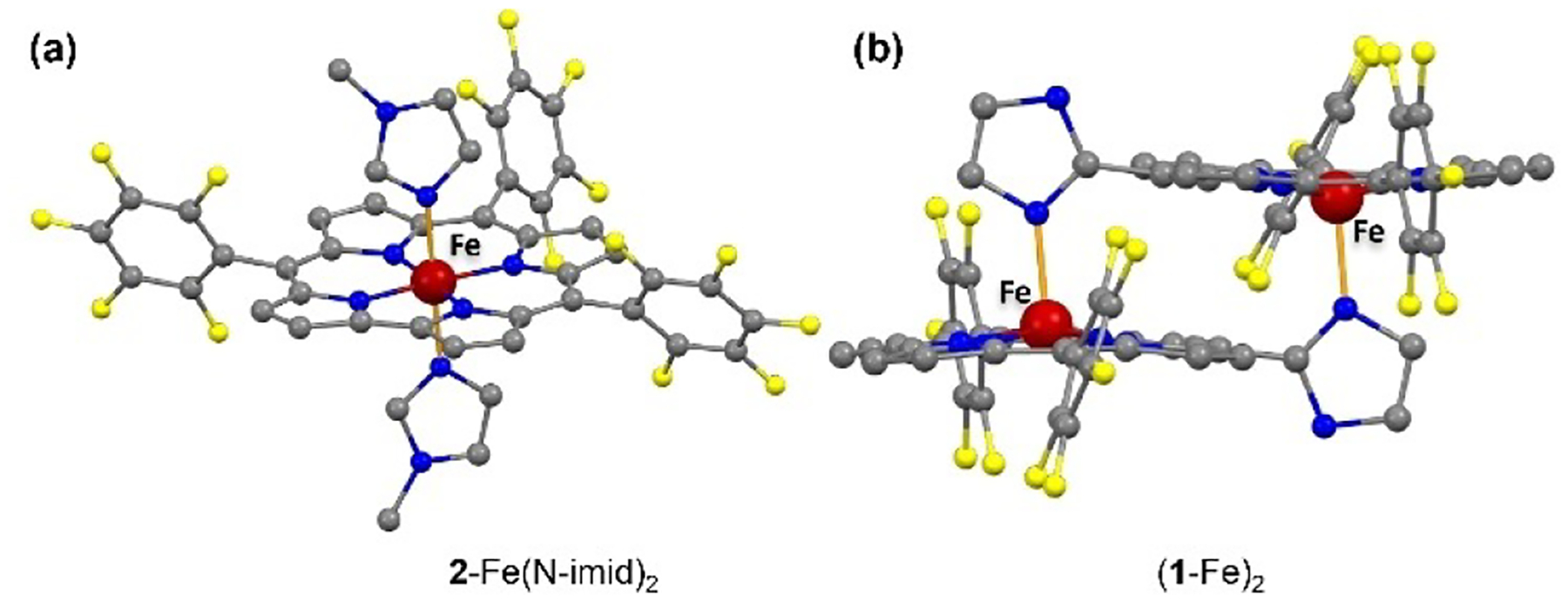
Ball and stick presentation of (a) **2**-Fe(N-imid)_2_ and (b) (**1**-Fe)_2_. Omitted are H-atoms and pyridines attached to (**1**-Fe)_2_ imidazole N–H units via hydrogen bonding.

**Figure 2.**
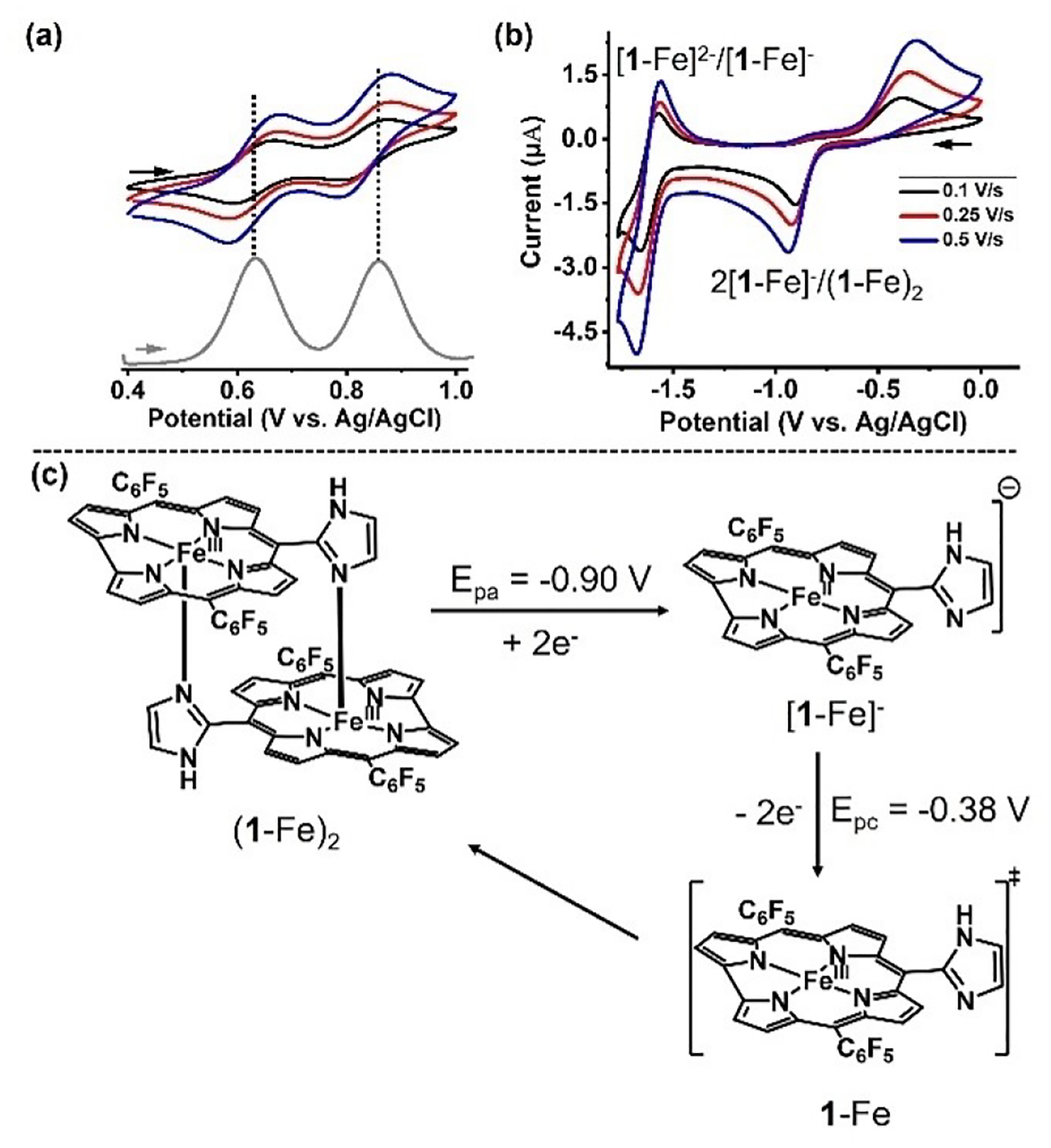
Voltammograms of 0.5 mM (**1**-Fe)_2_ (a & b) were measured in dry degassed PhCN solution containing 0.1 M TBAP under an argon atmosphere at 25°C. Spectra in black, red, and blue in (a) and (b) were recorded at scan rates of 0.1, 0.25, and 0.5 V/s, respectively. The square wave (grey bands in a) was scanned at 50 Hz. Proposed steps in the first reduction of (**1**-Fe)_2_ (c).

**Figure 3.**
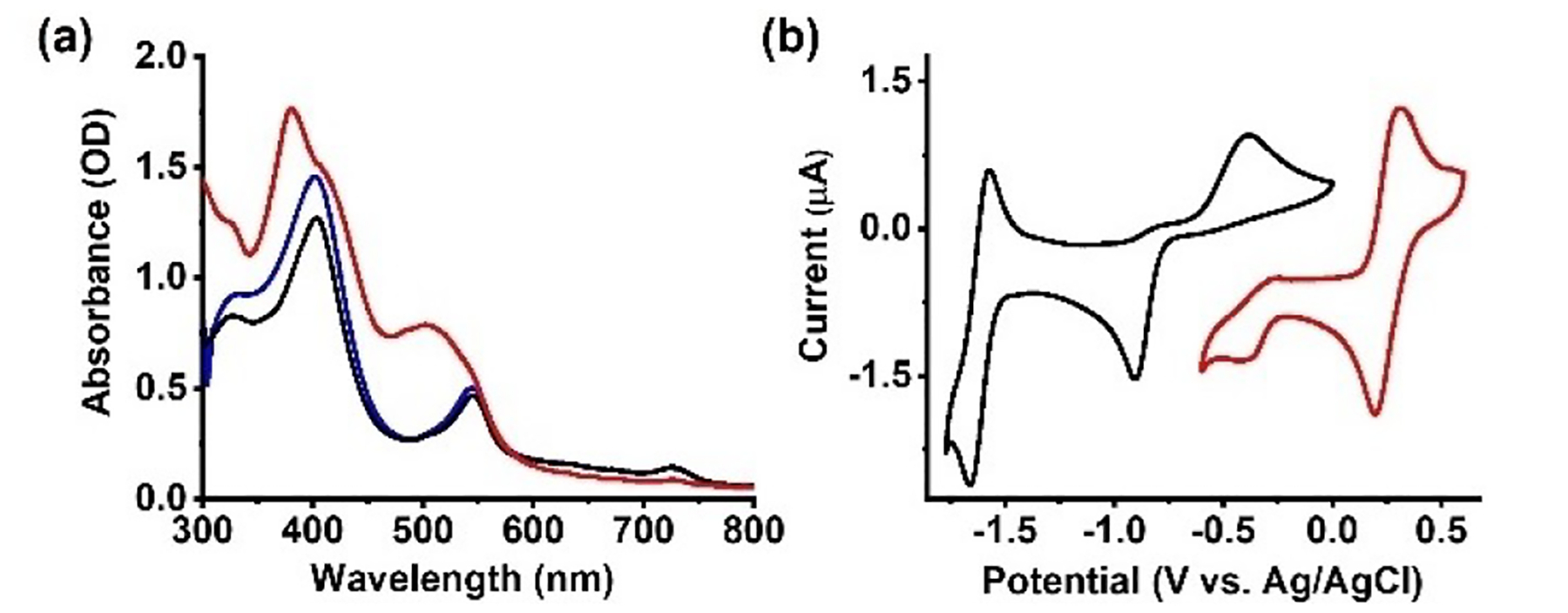
UV-vis spectra (a): (**1**-Fe)_2_ before (black) and after (red) addition of NO in THF solution. The original spectrum was restored after Ar-purging for 1 min (blue). Cyclic voltammograms (b): (**1**-Fe)_2_ before (black) and after (red) addition of NO(g) in dry degassed THF solution containing 0.1 M TBAP under an argon atmosphere at 25°C.

**Figure 4.**
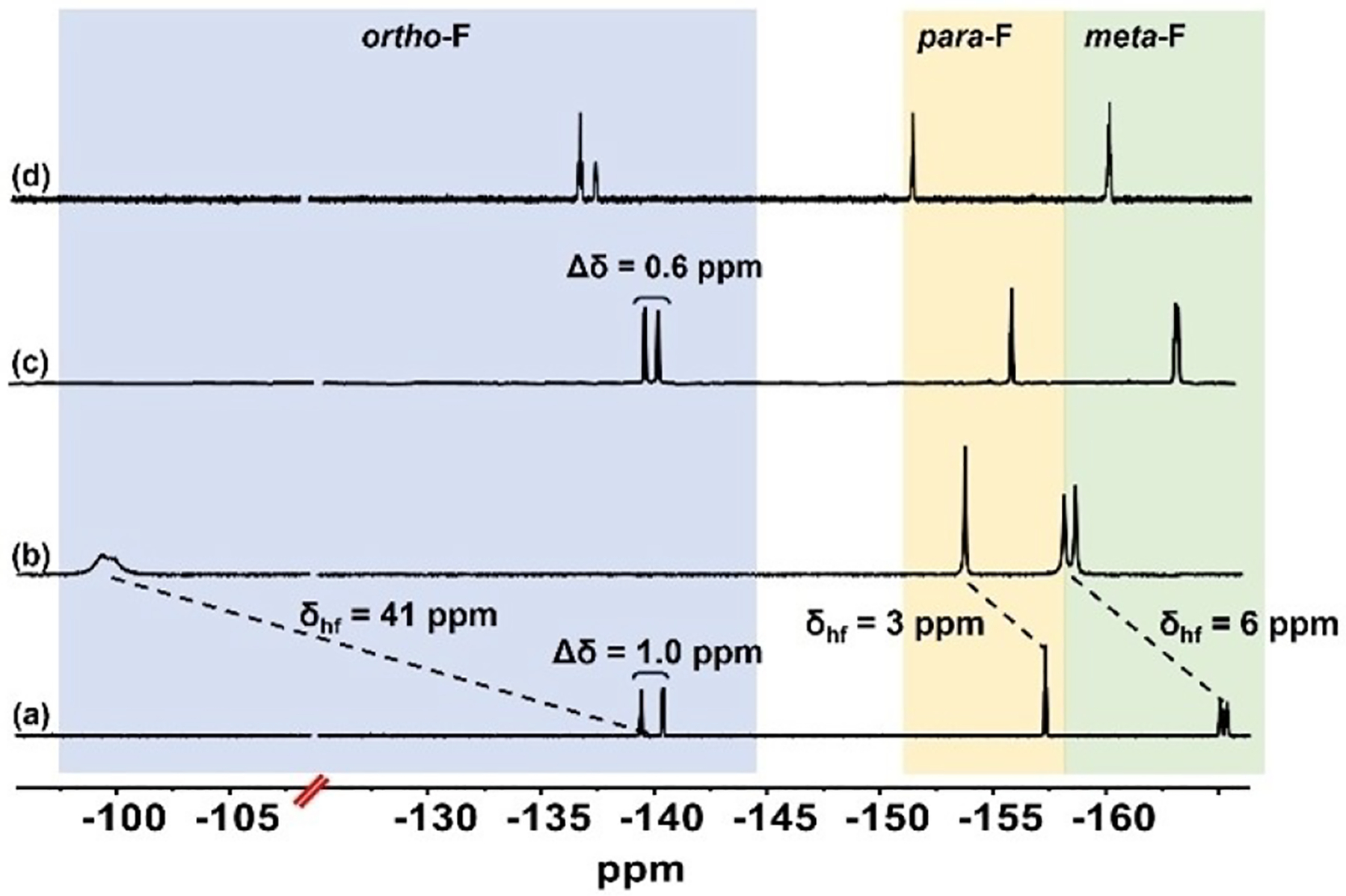
^19^F NMR spectra (THF-*d*_*8*_, 500 MHz) of (a) diamagnetic (**1**-Ga)_2_, (b) paramagnetic (**1**-Fe)_2_, (c) (**1**-Fe)_2_ upon treatment with NO(g), and (d) mononuclear **2**-Fe(NO). Δ is the difference between the chemical shifts and δ_hf_ is the paramagnetic/hyperfine shifts between (**1**-Fe)_2_ and the diamagnetic analogue (**1**-Ga)_2_. Half-height resonance widths are 1.19, 0.14, and 0.11 ppm for *ortho*-F, *meta*-F, and *para*-F, respectively.

**Figure 5.**
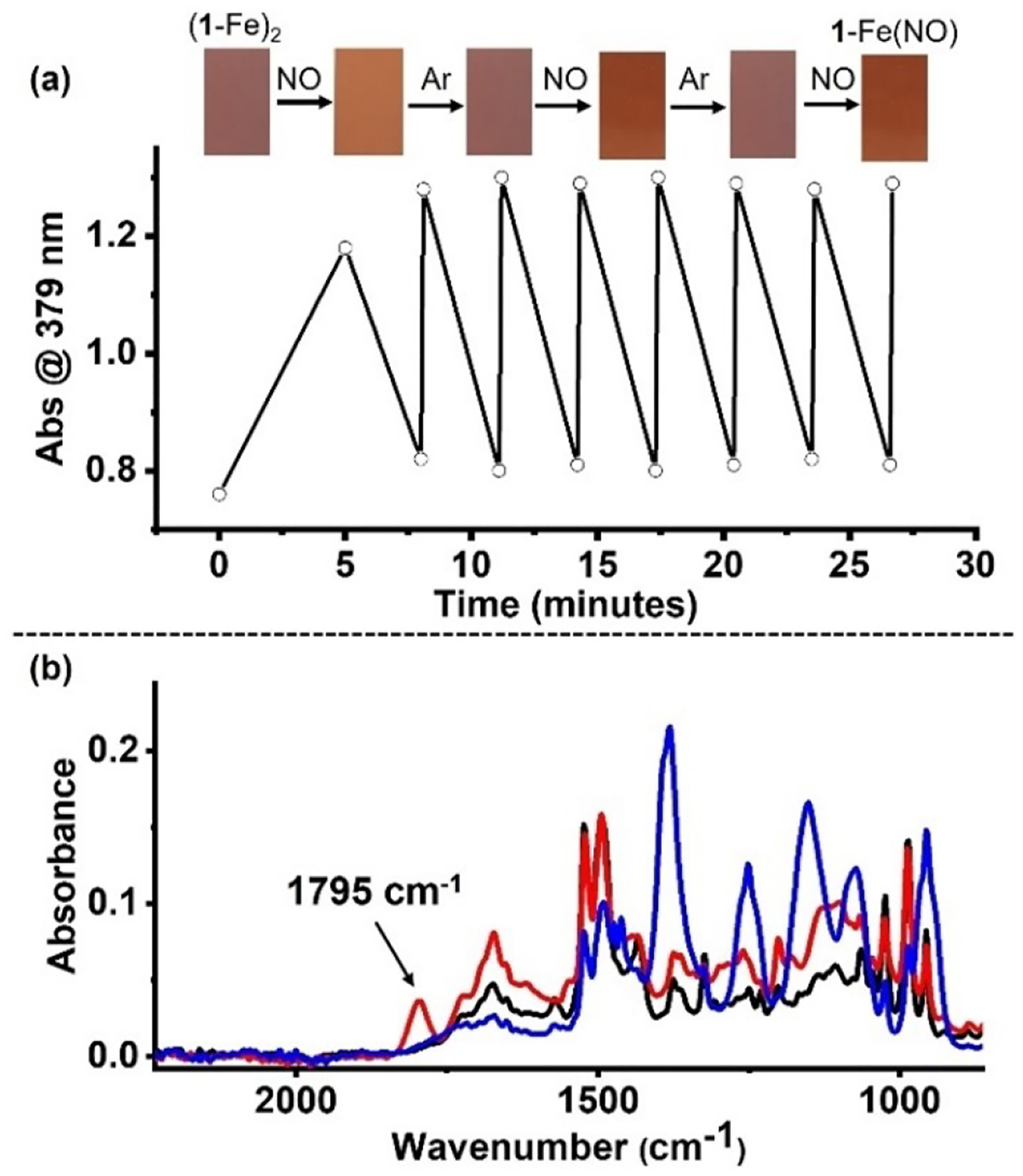
Absorption data collected at 379 nm supporting reversible formation of **1**-Fe(NO) (a): re-exposure to Ar releases NO, restoring (**1**-Fe)_2_. The images [top (a)] represent consecutive color changes of (**1**-Fe)_2_ solutions upon exposure to NO gas under an inert atmosphere. IR spectra of (**1**-Fe)_2_ (b): before (black) and after (red) NO purging the solution and casting a drop on the IR surface. Blowing Ar on the IR surface recovers the original spectrum (blue).

**Scheme 1.**
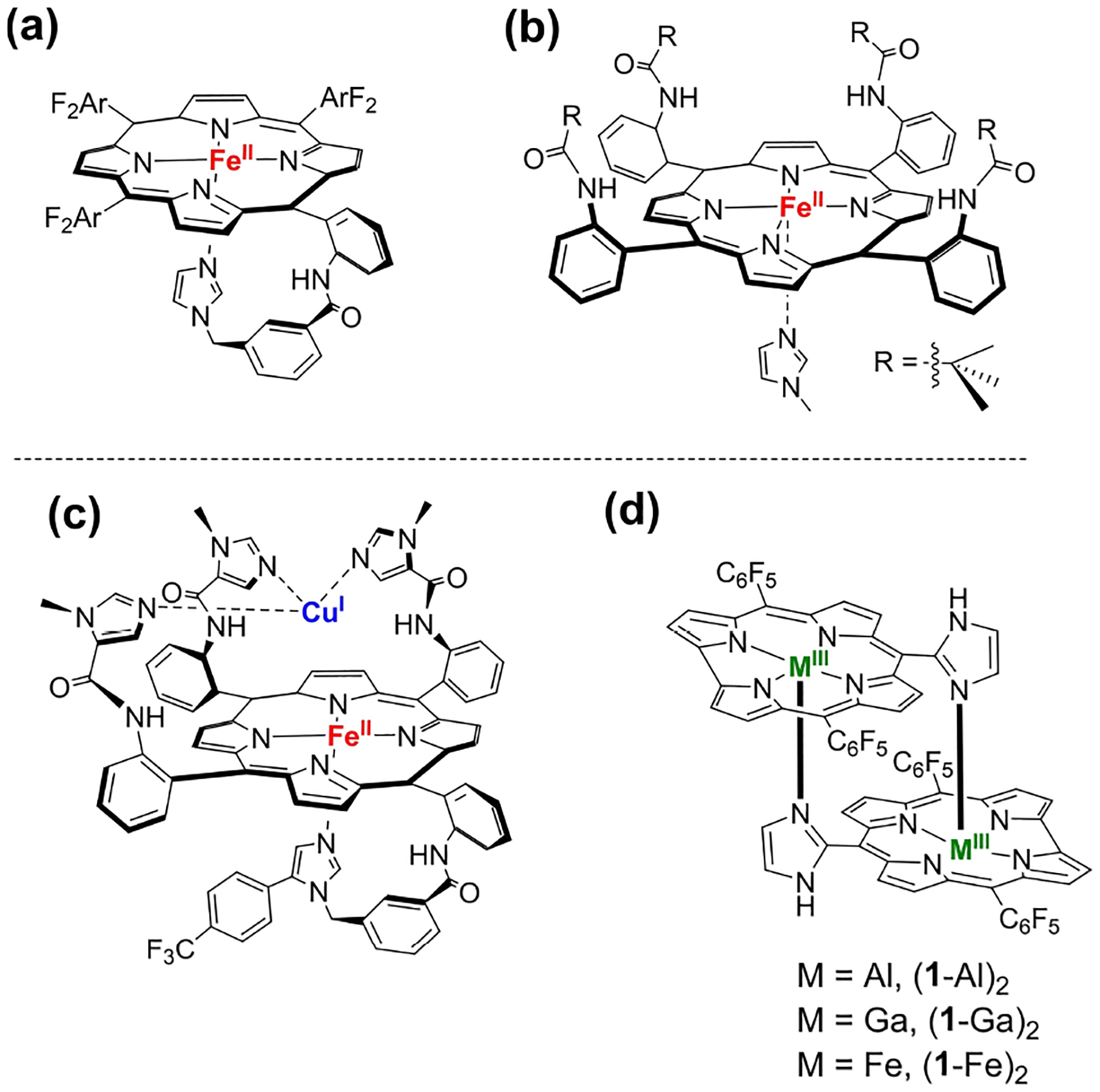
Iron porphyrins with an axial imidazole and a vacant axial binding site (a-c). Corrole dimers, M=Ga, Al, Fe (d).

## Data Availability

The data that support the findings of this study are available in the supplementary material of this article.
