# Supplementary material for: Reversible Reactions of Nitric Oxide with a Binuclear Iron(III) Nitrophorin Mimic
Source: Chemistry. Author manuscript; Available in PMC 2024 Sep 18. (PMC11410176; doi:10.1002/chem.202302860)
Supplement: supporting information [file NIHMS2022644-supplement-supporting_information.pdf]

Chemistry–A European Journal

Supporting Information

Reversible Reactions of Nitric Oxide with a Binuclear Iron(III) Nitrophorin Mimic

Vinay K. Sharma, Azad Saini, Natalia Fridman, Harry B. Gray,* and Zeev Gross*

Contents

Title	Page No.
**Experimental procedures.**	**3-4**
**General methods and instruments.**	**3-4**
**Scheme S1.** Synthesis of **2**-Fe(N-imid)$_2$.	**4**
**Scheme S2.** Synthesis of (**1**-Fe)$_2$.	**5**
**Scheme S3.** Synthesis of **2**-Fe(NO).	**5**
**Scheme S4.** Synthesis of **1**-Fe(NO).	**6**
**Table S1.** Selected geometrical parameters of iron amine complexes.	**7**
**Table S2.** Summary of the crystallographic data of **2**-Fe(N-imid)$_2$ and (**1**-Fe)$_2$.	**7-8**
**Figure S1.** ^{1}H (top) and ^{19}F (bottom) NMR spectra of (**1**-Fe)$_2$ in THF-*d*$_8$.	**9**
**Figure S2.** UV−vis spectral changes of (a) (**1**-Fe)$_2$ and (b) **2**-Fe(N-imid) with increasing concentration of N-methyl imidazole (N-imid) in PhCN at 298 K. The insets of (a & b) show the colors of the solutions at their start and end points. The corresponding conversion of (c) 5-coordinate **2**-Fe(N-imid) to 6-coordinate **2**-Fe(N-imid)$_2$ and (d) 5-coordinate dimer (**1**-Fe)$_2$ to 6-coordinate monomer **1**-Fe(N-imid)$_2$ upon addition of N-imid.	**10**
**Figure S3.** High-resolution observed mass (top) and low-resolution simulated (bottom) spectra of (**1**-Fe)$_2$.	**11**
**Figure S4.** Cyclic voltammogram of **2**-Fe(py)$_2$ (0.5 mM in PhCN containing 0.1M TBAP) measured at a scan rate of 100 mV/s under an argon atmosphere at 25 °C.	**12**
**Figure S5.** ^{1}H NMR spectrum of **1**-Fe(NO) in THF-*d*$_8$.	**12**
**Figure S6.** ^{1}H NMR spectrum of **2**-Fe(NO) in $CDCl_3$.	**13**
**Figure S7.** Cyclic voltammogram of **2**-Fe(py)$_2$ (1 mM) before (black) and after (red) addition of NO. The measurements were conducted at a scan rate of 100 mV/s in dry degassed THF solution containing 0.1 M TBAP under an argon atmosphere at 25 °C.	**13**
**Figure S8.** UV-vis spectra of **2**-Fe(py)$_2$ before (black) and after (red) addition of NO in THF solution.	**14**
**Figure S9.** IR spectrum of **2**-Fe(py)$_2$ (black) and **2**-Fe(NO) (red).	**14**
**Figure S10.** UV-vis spectra of **2**-Fe(N-imid)$_2$ before (black) and after (red) addition of NO in THF solution.	**15**

**Figure S11.** The EPR of (**1**-Fe)$_2$ in different solvents at 5 K (a) perpendicular mode and (b) parallel mode. Variable temperature magnetic susceptibility measured on microcrystalline samples in the range of 2-300K by using SQUID magnetometry for (**1**-Fe)$_2$ (c).	**16**

Experimental Procedures

General methods and instruments:

Commercially available chemicals were purchased from Sigma-Aldrich, Merck, and Chem Intel and used as received unless otherwise stated. Analytical reagent (AR) grade solvents were used for the reactions while spectroscopic grade solvents were used for photophysical and electrochemical properties. Pyrrole was subjected to filtration on a column packed with neutral aluminum oxide before use, while the rest of the reagents were used without further purification. Unless mentioned separately, synthesis was performed at ambient conditions. **1**-H_3,[17] and **2**-Fe(py)$_2$[18] were synthesized according to literature reports. Column chromatography was performed on silica gel (Kieselgel 60, 230–400 mesh). Nitrosyl Oxide (NO) gas was prepared from the reaction of $NaNO_2$ and 4N H_2SO_4 followed by purification via NaOH pellet. One gram of solid $NaNO_2$ was taken in a three-neck round bottom flask. Subsequently, an aqueous solution of H_2SO_4 was then added dropwise under a flow of nitrogen gas. It is crucial to ensure the complete removal of any residual oxygen within the flask and accompanying tubes. To obtain a highly purified NO gas, the evolved gas was subjected twice through a 30% w/w aqueous solution of NaOH, followed by absorption on NaOH pellets. The concentration of NO gas was modulated by the concurrent flow of N_2 gas. In all experiments that include NO gas purging, the NO gas used was in its fresh state. Absorption spectra were measured of synthesized corroles were recorded on Agilent Technologies Cary 8454 UV-vis spectrophotometer. Quartz cuvettes of 1.0 cm thickness were used to measure the samples. ^{1}H and ^{19}F NMR spectra were recorded on a Bruker Avance III 400/500 MHz spectrometer equipped with a 5 mm diameter of the sample and matching with a broad-band probe (BBFO) by z-gradients. The 400 NMR operates at a frequency of 400.4 MHz for proton (^{1}H) nuclei and 377 MHz for fluorine (^{19}F) nuclei whereas 500 NMR operates at a frequency of 500 MHz for ^{1}H nuclei and 471 MHz for ^{19}F nuclei. Chemical shifts are reported in ppm. THF-d_8 (δ = 1.72, 3.58) and $CDCl_3$ (δ = 7.26) were applied as an internal standard. MesteRenova v6.0.2-5475 software was used for the analysis of NMR spectra. High-resolution mass spectra for the compounds were performed on a Bruker MaXis Impact mass spectrometer, using APCI (atmospheric pressure chemical ionization) direct

probe in either positive or negative mode. Electrochemical measurements were performed on a 3-electrode system using EMStat3+ electrochemical system and the glassy carbon (working electrode), Ag/AgCl (reference electrode), and platinum wire (counter electrode). The CV measurements were performed in benzonitrile (PhCN) solutions (HPLC grade) containing 0.1 M tetrabutylammonium perchlorate (TBAP) as electrolyte and 0.5 mM iron complexes at ambient temperature with different (100-250 mV/s) applied scan rates. However, CV measurements of iron nitrosyl complexes were performed in tetrahydrofuran (THF) containing 0.1 M TBAP as electrolyte and 1 mM iron nitrosyl complexes at ambient temperature with 100 mV/s applied scan rates. The iron nitrosyl complex of $(\mathbf{1}\text{-Fe})_2$ was prepared *in situ* during electrochemical measurement upon purging the NO gas into the cell. Single crystals immersed in Paratone-N oil were quickly fished with a glass rod and mounted on a Kappa CCD diffractometer under a cold stream of nitrogen at 200 K. Data collection was carried out with monochromated Mo Kα radiation using φ and ω scans to cover the Ewald sphere.[45] Accurate cell parameters were obtained with complete collections of intensities, and these were corrected in the usual way.[46] The structures were solved by SHELXS-97 direct methods[47] and refined by the SHELXL-97 program package. The atoms were refined anisotropically. Hydrogen atoms were calculated using the Riding model. The measurement of infrared spectra was conducted utilizing a Bruker Tensor 27 spectrometer that was equipped with an attenuated total reflectance (ATR) accessory. Background measurements were taken in the air and subsequently subtracted from the spectra for each measurement. The spectra were subjected to an averaging process of 64 scans and were acquired under ambient conditions at a temperature of 22°C. Magnetic susceptibility data were collected using a Quantum Design MPMS3 SQUID magnetometer over the temperature range 2 to 300 K. Data were collected over the temperature range of 2 to 300 K, by using applied magnetic fields of 1 T and corrected for diamagnetism using Pascal's constant. Continuous wave X-band EPR spectra were obtained on a Bruker EMX spectrometer on 1.0-3.0 mM solutions prepared as frozen solutions in 2-MeTHF, THF, PhCN, or propionitrile. X-band HYSCORE experiments were performed using a Bruker MS5 resonator. Temperature control was achieved using an ER 4118HV-CF5-L Flexline CryogenFree VT cryostat manufactured by ColdEdge equipped with an Oxford Instruments Mercury ITC temperature controller. The samples were input into a capillary micropipette sealed at one end and inserted into an outer quartz tube of 3mm (ID). Then the tubes were put into the cryostat and measured.

Synthesis of iron corroles

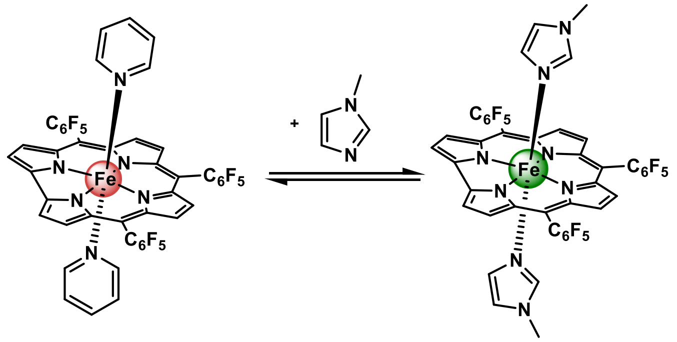

Scheme S1. Synthesis of **2**-Fe(N-imid)$_2$.

Synthesis of **2**-Fe(N-imid)$_2$. **2**-Fe(py)$_2$ (4.3 mg, 0.005 mmol) was dissolved in THF (1ml) followed by the addition of 1 drop of N-methyl imidazole and stirring for 5 min at room temperature. The color of the solution was changed from reddish brown to dark green. **2**-Fe(N-imid)$_2$ (4.8 mg, 0.005 mmol) was isolated and crystallized from a mixture of CH_2Cl_2/heptane/N-methyl imidazole. The crop of green crystals was collected after 5 days in 95% yields. UV-Vis (THF) λ_{max} (relative intensities): 406 (1.0), 547 (0.2), 707 (0.07) nm.

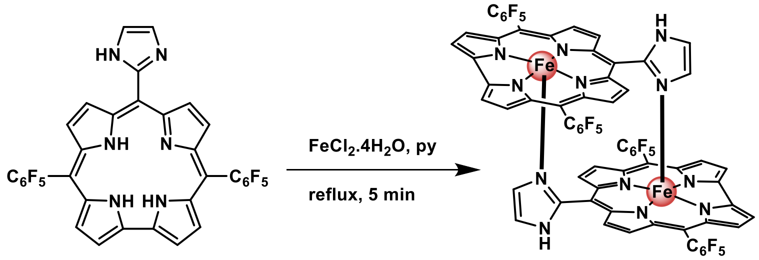

Scheme 2. Synthesis of (**1**-Fe)$_2$.

Synthesis of (**1**-Fe)$_2$. **1**-H_3 (80 mg, 0.1mmol) was dissolved in pyridine (30ml) followed by the addition of $FeCl_2$ (250 mg, 2 mmol); then the reaction mixture was reflux at 80 °C under N_2 for 5 minutes. The progress of the reaction was followed by TLC (Silica, 100 % Et_2O). After that, the solvent was evaporated and the impurities were removed by flash chromatography (silica, 100 % Et_2O), followed by recrystallization from pyridine. (**1**-Fe)$_2$ was obtained as red crystals (127 mg, 85% yield). ^{1}H-NMR (500 MHz, THF-d_8): δ (ppm) = 64.80, 10.17, -5.91, -66.04, -99.33. ^{19}F NMR (471 MHz, THF-d_8): δ(ppm) = -99.5 (*ortho*-F), -154.02 (*para*-F), -158.42 (*meta*-F), -158.94 (*meta*-F). High-resolution mass spectrum (APCI, Positive mode) m/z:

Calculated for $C_{68}H_{23}F_{20}Fe_2N_{12}$: 1499.0547; Observed: 1499.0582 (100) [M+H]. UV-vis (THF) λ_{max} (relative intensities): 327 (0.61), 403 (1.0), 547 (0.35), 726 (0.08) nm.

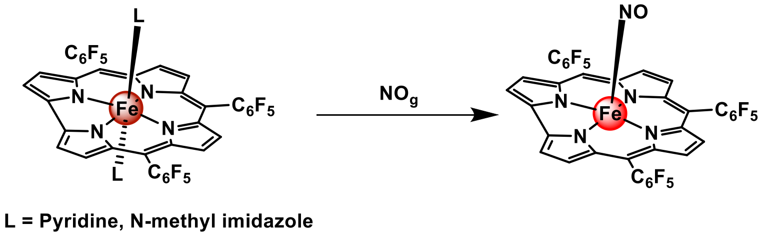

Scheme 3. Synthesis of **2**-Fe(NO).

Synthesis of **2**-Fe(NO). **2**-Fe(py)$_2$ (20 mg) was dissolved in dichloromethane (10 ml) under N_2 atmosphere at room temperature. Purified colorless NO (g) was gently purged into the solution. The progress of the reaction was followed by TLC (Silica/Et_2O) and UV-vis changes. After a few minutes, the color of the reaction mixture changed from reddish brown to deep red indicating the product formation. The product was purified using column chromatography and isolated in good yield. ^{1}H NMR (400 MHz, THF-d_8) δ (ppm) = 8.18 (d, J = 4.7 Hz, 2H), 7.76 (d, J = 4.6 Hz, 2H), 7.51 (d, J = 4.7 Hz, 2H), 7.44 (d, J = 4.7 Hz, 2H). ^{19}F NMR (377 MHz, THF-d_8) δ -137.11 (t, J = 28.4 Hz, 4H), -137.79 (d, J = 19.5 Hz, 2H), -151.75 (dd, J = 25.6, 16.6 Hz, 3H), -160.38 (dd, J = 45.1, 22.4 Hz, 6H). UV-vis (THF) λ_{max} (relative intensities): 379 (1.0), 540nm (0.15) nm. IR: υ_{NO} = 1800 cm^{-1}.

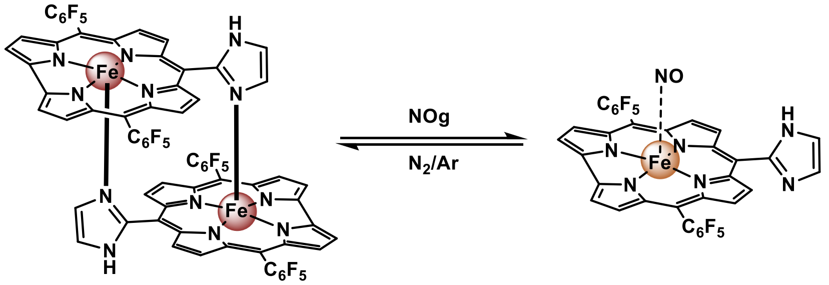

Scheme 4. Synthesis of **1**-Fe(NO).

Synthesis of **1**-Fe(NO). A solution of (**1**-Fe)$_2$ (7 mg) was prepared by dissolving it in 0.5 ml of THF-d_8 in a J-young NMR tube. The solution was then purged with N_2 for a duration of 1-2 minutes. Subsequently, the mixture was then slowly purged with freshly prepared NO(g). The immediate color change was observed from brown to orangish red. [Caution: Excess of NO(g) might lead to nitration of the molecule]. The product was not isolated due to loosely bound NO. However, the product was characterized *in situ* by NMR UV-Vis, CV, and IR, which provided evidence for the formation of the product. ^{1}H NMR (500 MHz, THF-d_8) δ (ppm) = 8.26 (d, J = 3.7 Hz, 2H), 8.06 (s, 2H), 7.94 (d, J = 3.5 Hz, 2H), 7.72 (s, 2H), 7.48 (s, 2H). ^{19}F

NMR (471 MHz, THF-d_8) δ (ppm) = -139.51 (d, J = 22.8 Hz), -140.10 (d, J = 22.6 Hz), -155.82 (t, J = 20.6 Hz), -163.02 – -163.33 (m). UV-vis (THF) λ_{max} (relative intensities): 380 (1.0), 410 (0.85), 505 (0.45) nm. IR: υ_{NO} = 1795 cm^{-1}.

X-ray data

	(**1**-Fe)$_2$ Core I	Core II	**2**-Fe(py)$_2$	**2**-Fe(N-imid)$_2$
M−N$_c$[Å]^a	1.894 (7)	1.892 (9)	1.865(5) – 1.923(5)	1.847(5) – 1.889(5)
M−N$_{N\text{-imid/py}}$ (Å)^b	2.085 (1)	2.113 (9)	2.028(5) – 2.032(5)	1.984(4) – 1.993(4)
$\Delta^M{}_4$(Å)^c	0.388	0.388	0.001(1)	0.013
M···M (Å)^d	5.988		**	**
MPS (Å)^f	2.935		**	**

Table S1. Selected geometrical parameters of iron amine complexes. [a] Average distance of metal to the inner N atoms of the corrole. [b] Average distance between the metal center(s) and the axial pyridines/imidazoles. [c] Displacement of metal from the plane of the N4 corrole core. [d] Metal to metal distance. [f] Average distance between the two planes of the N_4 corrole cores.

**Crystal data**	**2**-Fe(N-imid)$_2$	(**1**-Fe)$_2$
**Empirical formula**	$C_{45}H_{20}F_{15}FeN_8$	$C_{88}H_{42}F_{20}Fe_2N_{16}$
**Formula weight**	1013.54	1815.07
**Temperature (K)**	140.15	200.15
**Wavelength (Å)**	0.71073	0.71073
**Crystal system,**	monoclinic	monoclinic
**space group**	C2/c	P2$_1$/n
**a (Å)**	26.30(2)	11.074(3)
**b (Å)**	27.01(2)	25.399(7)
**c (Å)**	40.96(3)	26.811(7)
**alpha**	90	90

**beta**	101.93(2)	91.959(7)
**gamma**	90	90
**Volume (Å^3)**	28471(4)	7537(4)
**Z**	24	4
**Calculated density (mg/m^3)**	1.419	1.600
**Absorption coefficient (mm^{-1})**	0.417	0.497
**F(000)**	12168	3656.0
**Crystal size (mm)**	0.33 × 0.12 × 0.12	0.27 × 0.12 × 0.09
**2Theta range**	2.268 – 49.692	3.04 – 47.216
**Reflection collected/unique**	96478 / 23581	18650 / 6892
**Rint**	0.0819	0.1127
**Completeness (%)**	98.1	98.4
**Absorption correction**	semi-empirical	semi-empirical
**Data/restraints/ parameters**	23581/3361/2216	6892/60/973
**Goodness-of-fit on F^2**	0.984	0.927
**R1, wR2 [I>2sigma(I)]**	0.0606, 0.1519	0.0975, 0.1998
**R1, wR2 (all data)**	0.1585, 0.1967	0.2605, 0.2695
**Largest diff. peak and hole**	1.06 /-0.57	1.37 / -0.93
**Diffractometer**	Bruker Apex II	Bruker Apex II

Table S2. Summary of the crystallographic data of **2**-Fe(N-imid)$_2$ and (**1**-Fe)$_2$.

NMR spectrum

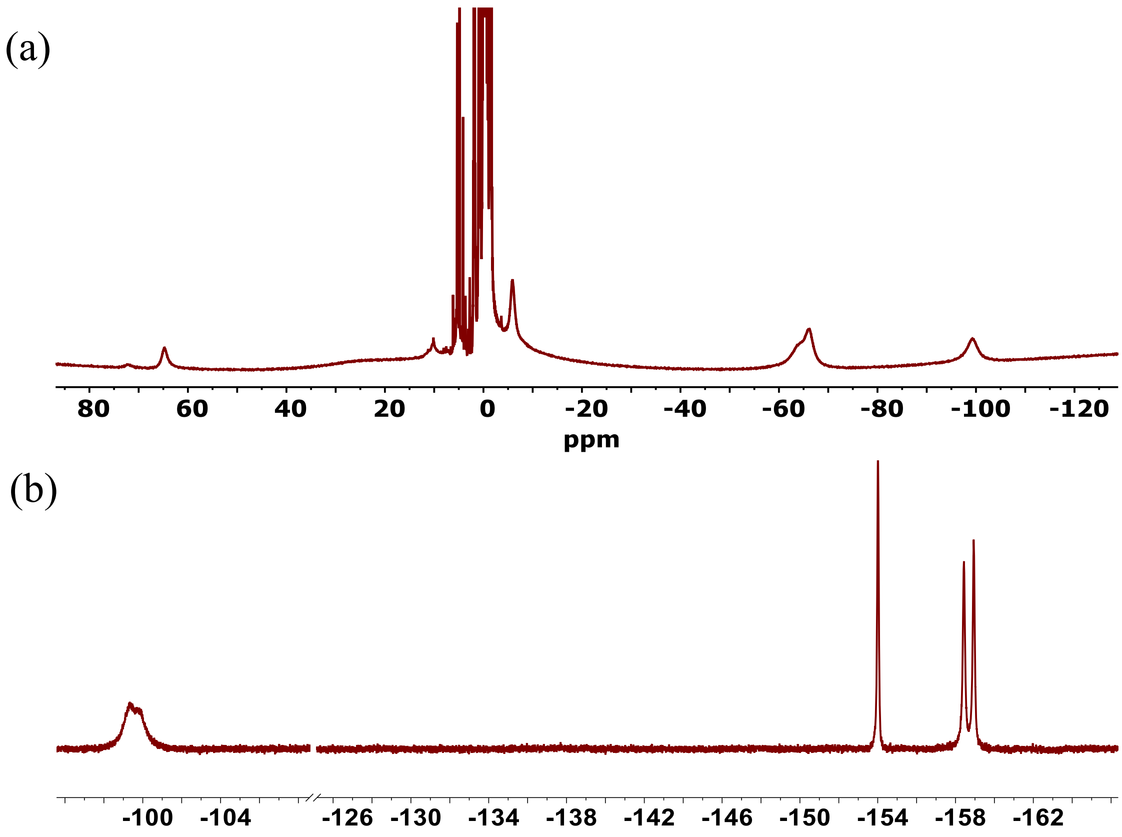

Figure S1. ^{1}H (top) and ^{19}F (bottom) NMR spectra of (**1**-Fe)$_2$ in THF-d_8.

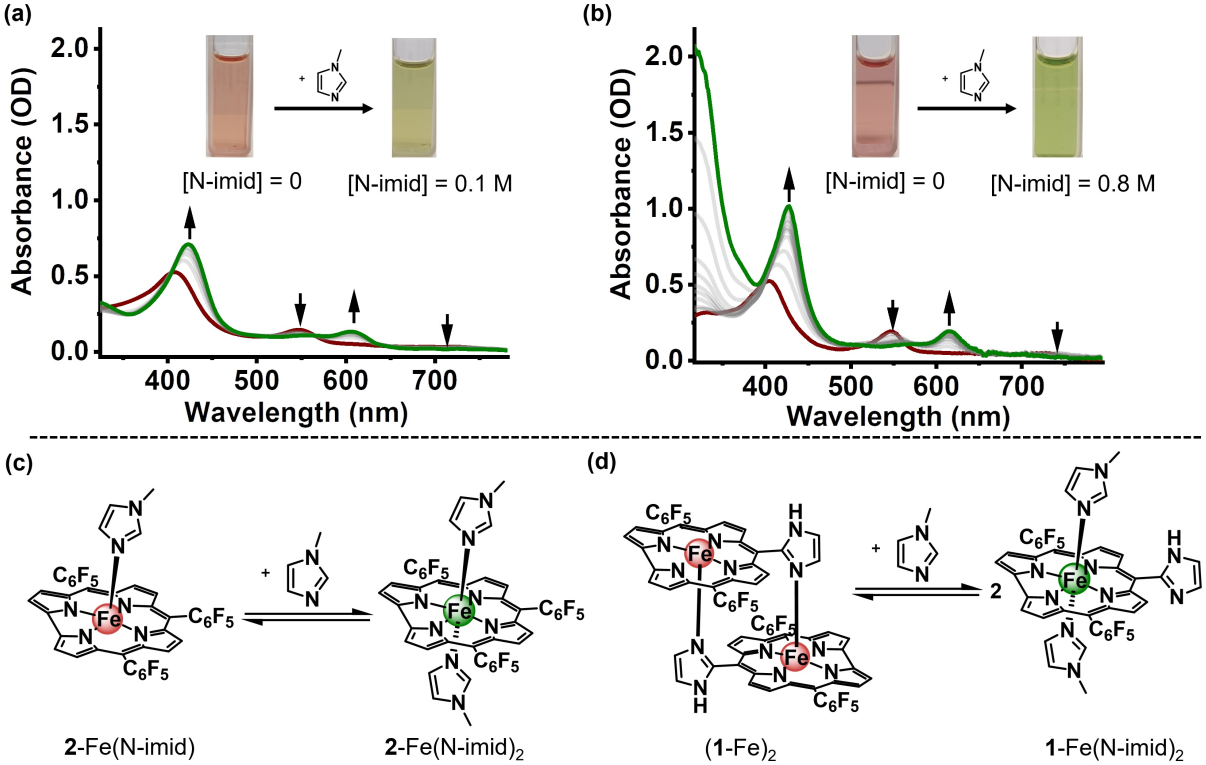

Figure S2. UV−vis spectral changes of (a) (**1**-Fe)$_2$ and (b) **2**-Fe(N-imid) with increasing concentration of N-methyl imidazole (N-imid) in PhCN at 298 K. The insets of (a & b) show the colors of the solutions at their start and end points. The corresponding conversion of (c) 5-coordinate **2**-Fe(N-imid) to 6-coordinate **2**-Fe(N-imid)$_2$ and (d) 5-coordinate dimer (**1**-Fe)$_2$ to 6-coordinate monomer **1**-Fe(N-imid)$_2$ upon addition of N-imid.

High-resolution mass spectrum

Display Report

Analysis Info

Analysis Name	D:\Data\Gross\Gr_4263000001.d	Acquisition Date	07/05/2023 12:46:57
Method	APCI_pos_SolidProbe.m	Operator	Larisa Panz
Sample Name	Fe dimer	Instrument	maXis impact 282001.00128
Comment			

Acquisition Parameter

Source Type	APCI	Ion Polarity	Positive	Set Nebulizer	1.2 Bar
Focus	Active	Set Capillary	3000 V	Set Dry Heater	300 °C
Scan Begin	50 m/z	Set End Plate Offset	-500 V	Set Dry Gas	1.5 l/min
Scan End	2000 m/z	Set Charging Voltage	2000 V	Set Divert Valve	Source
		Set Corona	5000 nA	Set APCI Heater	200 °C

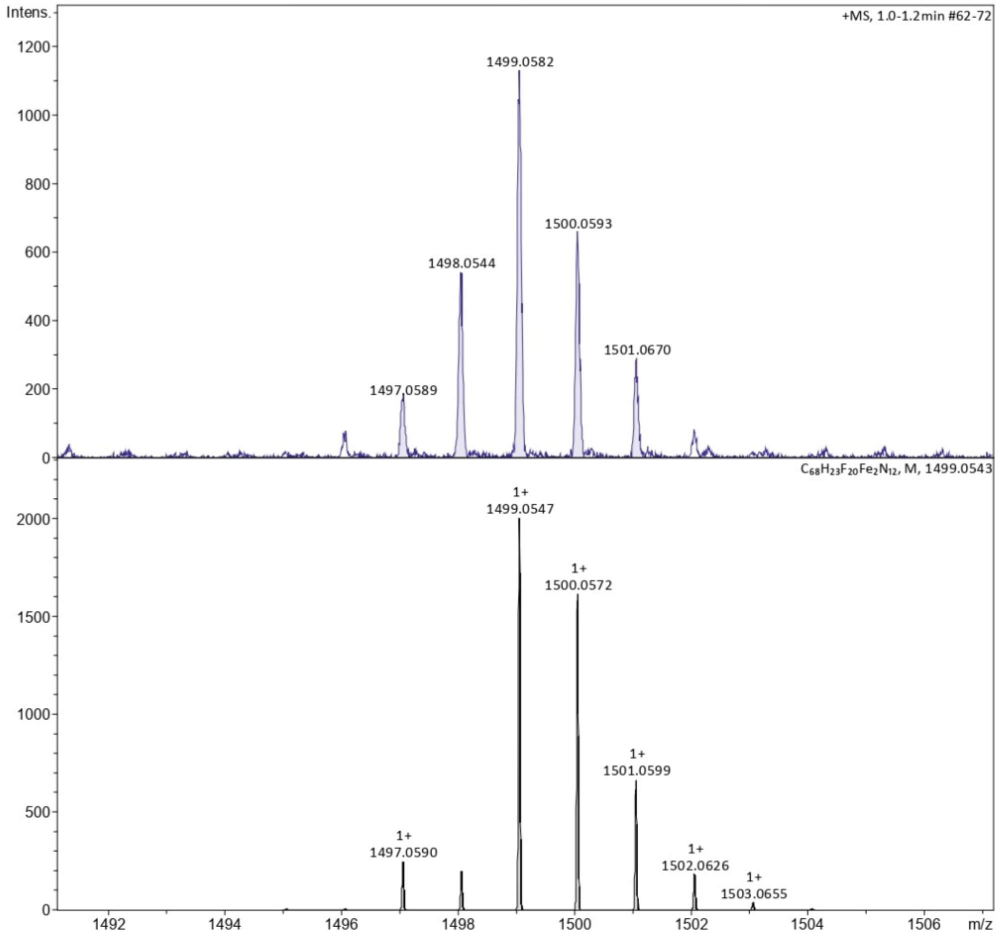

Gr_4263000001.d

Bruker Compass DataAnalysis 4.2 printed: 07/05/2023 13:01:47 by: Larisa Panz Page 1 of 1

Figure S3. High-resolution observed mass (top) and low-resolution simulated (bottom) spectra of (**1**-Fe)$_2$.

Cyclic voltammogram

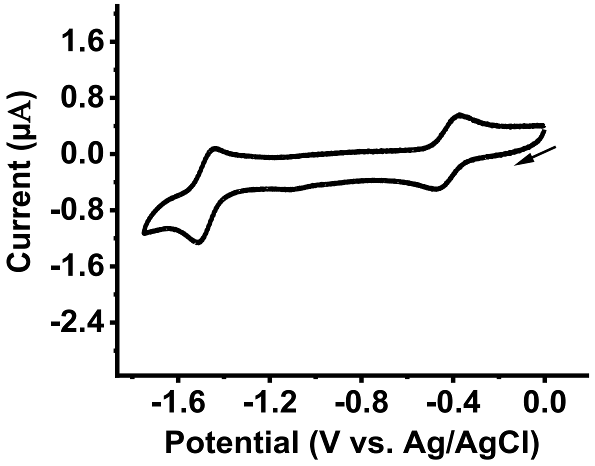

Figure S4. Cyclic voltammogram of **2**-Fe(py)$_2$ (0.5 mM in PhCN containing 0.1M TBAP) measured at a scan rate of 100 mV/s under an argon atmosphere at 25 °C.

^{1}H NMR spectrum

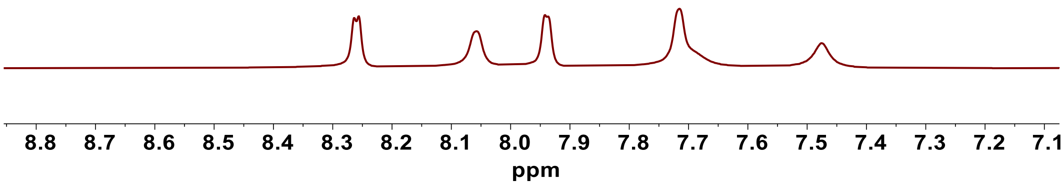

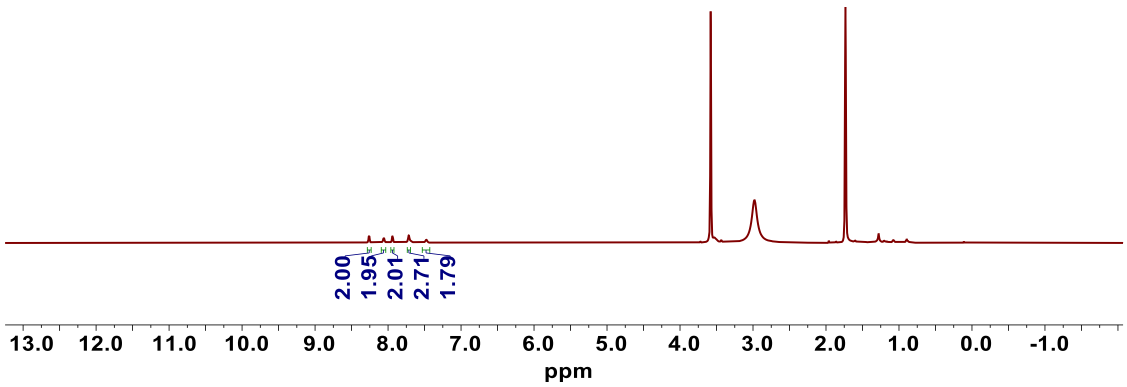

Figure S5. ^{1}H NMR spectrum of **1**-Fe(NO) in THF-d_8.

^{1}H NMR spectrum

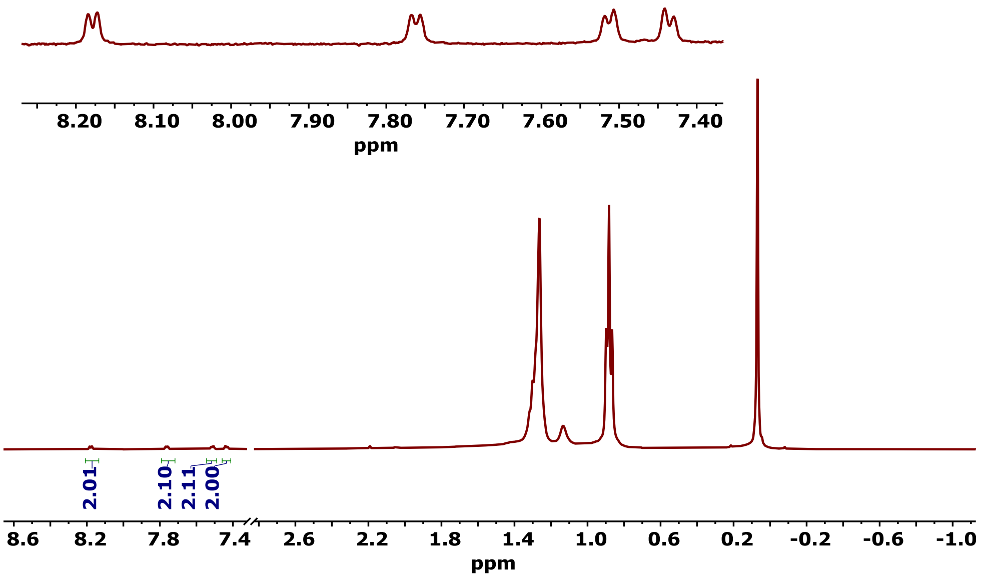

Figure S6. ^{1}H NMR spectrum of **2**-Fe(NO) in THF-d_8.

Cyclic voltammograms

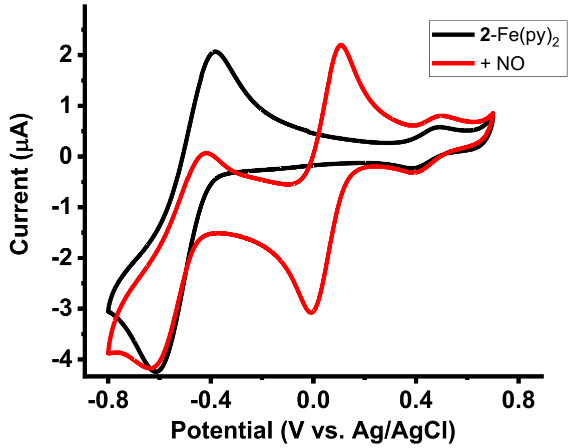

Figure S7. Cyclic voltammogram of **2**-Fe(py)$_2$ (1 mM) before (black) and after (red) addition of NO. The measurements were conducted at a scan rate of 100 mV/s in dry degassed THF solution containing 0.1 M TBAP under an argon atmosphere at 25 °C.

UV-vis spectra

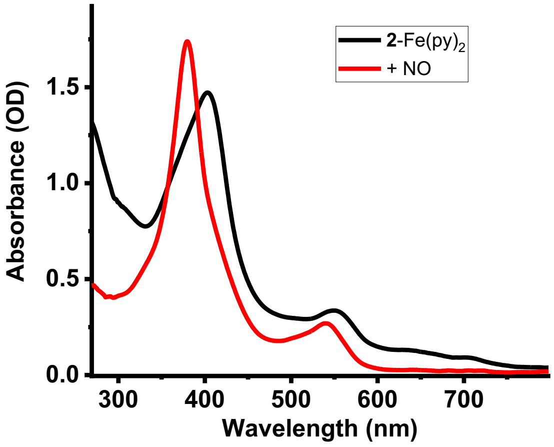

Figure S8. UV-vis spectra of **2**-Fe(py)$_2$ before (black) and after (red) addition of NO in THF solution.

IR spectra

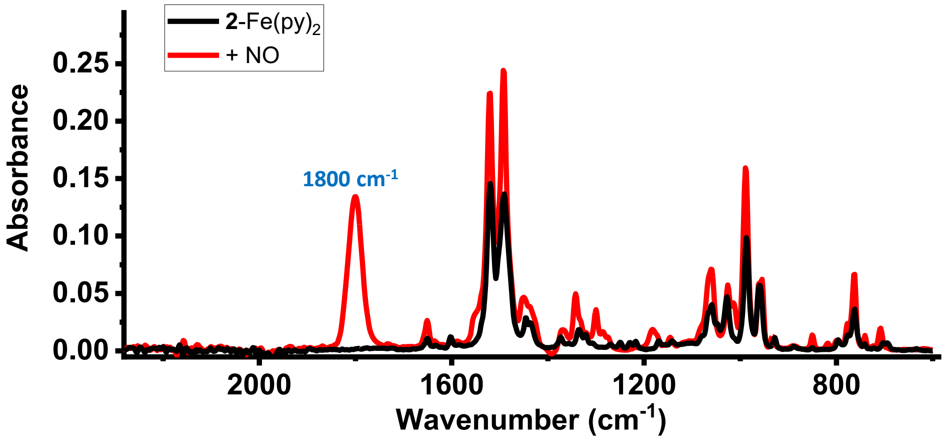

Figure S9. IR spectrum of **2**-Fe(py)$_2$ (black) and **2**-Fe(NO) (red).

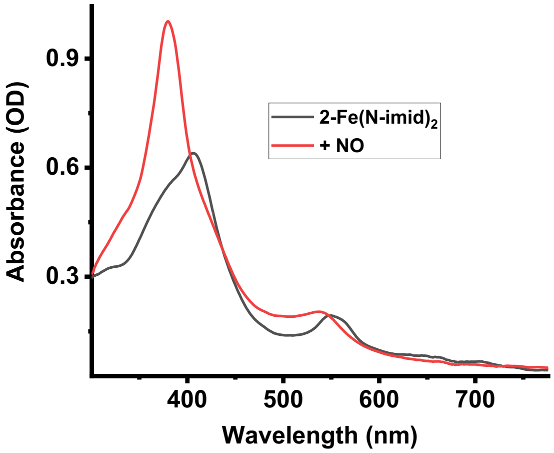

Figure S10. UV-vis spectra of **2**-Fe(N-imid)$_2$ before (black) and after (red) addition of NO in THF solution.

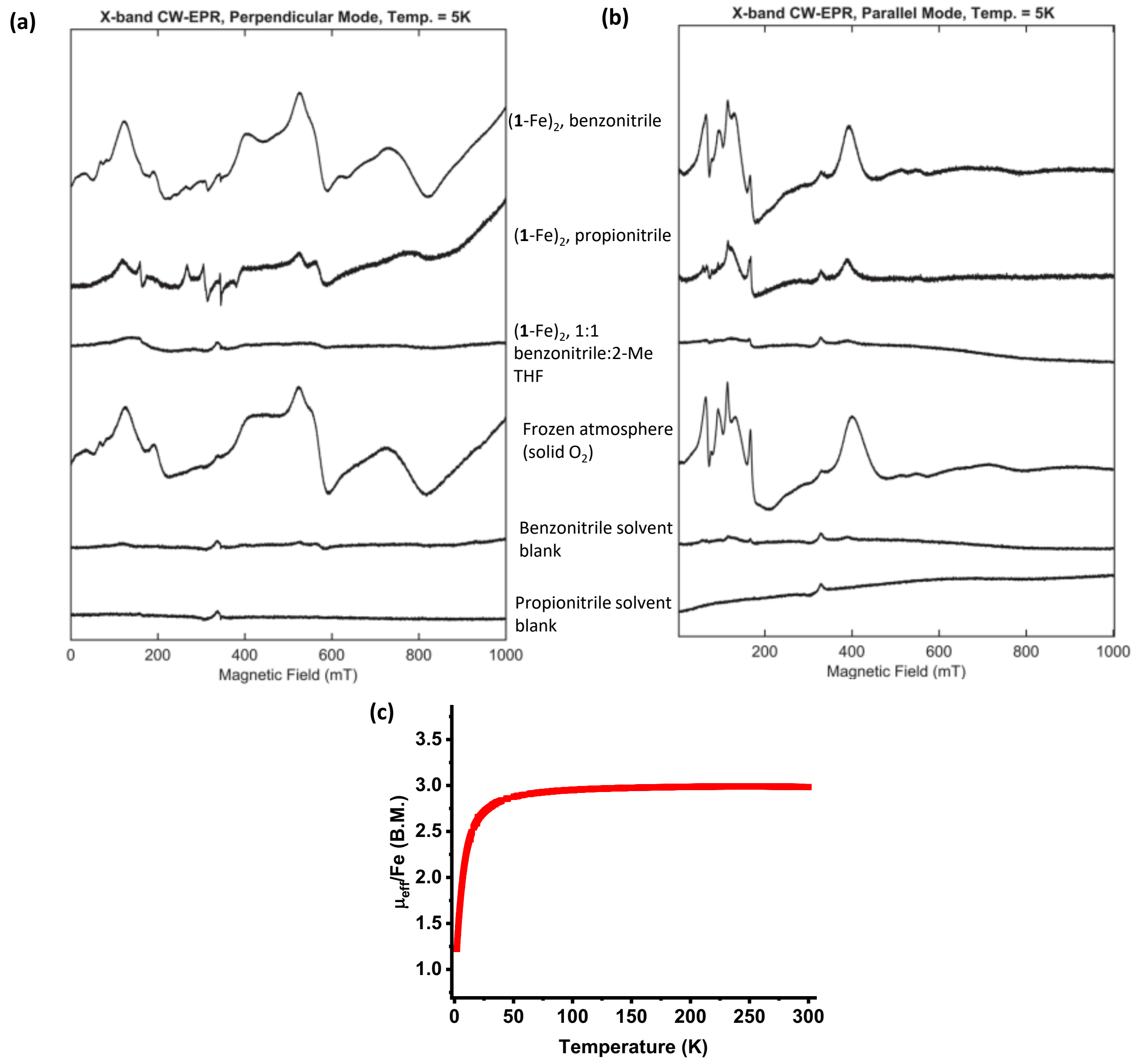

Figure S11. The EPR of (**1**-Fe)$_2$ in different solvents at 5 K (a) perpendicular mode and (b) parallel mode. Variable temperature magnetic susceptibility measured on microcrystalline samples in the range of 2-300K by using SQUID magnetometry for (**1**-Fe)$_2$ (c).
